# Two-dimensional multibit optoelectronic memory with broadband spectrum distinction

**DOI:** 10.1038/s41467-018-05397-w

**Published:** 2018-07-27

**Authors:** Du Xiang, Tao Liu, Jilian Xu, Jun Y. Tan, Zehua Hu, Bo Lei, Yue Zheng, Jing Wu, A. H. Castro Neto, Lei Liu, Wei Chen

**Affiliations:** 1 Department of Chemistry, National University of Singapore, Singapore, 117543 Singapore; 2 Centre for Advanced 2D Materials and Graphene Research Centre, National University of Singapore, 6 Science Drive 2, Singapore, 117546 Singapore; 3 Department of Physics, National University of Singapore, Singapore, 117542 Singapore; 4 State Key Laboratory of Luminescence and Applications, Changchun Institute of Optics, Fine Mechanics and Physics, Chinese Academy of Sciences, No. 3888 Dongnanhu Road, Changchun, 130033 People’s Republic of China; 5 Institute of Materials Research and Engineering (IMRE), 2 Fusionopolis Way, Singapore, 138634 Singapore; 6 National University of Singapore (Suzhou) Research Institute, 377 Lin Quan Street, Suzhou Industrial Park, Jiang Su, 215123 China

## Abstract

Optoelectronic memory plays a vital role in modern semiconductor industry. The fast emerging requirements for device miniaturization and structural flexibility have diverted research interest to two-dimensional thin layered materials. Here, we report a multibit nonvolatile optoelectronic memory based on a heterostructure of monolayer tungsten diselenide and few-layer hexagonal boron nitride. The tungsten diselenide/boron nitride memory exhibits a memory switching ratio approximately 1.1 × 10^6^, which ensures over 128 (7 bit) distinct storage states. The memory demonstrates robustness with retention time over 4.5 × 10^4^ s. Moreover, the ability of broadband spectrum distinction enables its application in filter-free color image sensor. This concept is further validated through the realization of integrated tungsten diselenide/boron nitride pixel matrix which captured a specific image recording the three primary colors (red, green, and blue). The heterostructure architecture is also applicable to other two-dimensional materials, which is confirmed by the realization of black phosphorus/boron nitride optoelectronic memory.

## Abstract (web summary)

Continued device miniaturization and feasibility of integrating two-dimensional materials into circuits have enabled flexible and transparent optoelectronic memories. Here, the authors show a WSe_2_–hBN-based heterostructure memory with switching ratio of ~1.1 × 10^6^, ensuring over 128 distinct storage states and retention time of ~4.5 × 10^4^ s.

## Introduction

Optoelectronic memories have attracted tremendous attention owing to its unique capability of accumulating and releasing photo-generated carriers under electrical stress and light irradiation^1–4^. This advantage enables the great potential of optoelectronic memories in image capturing, confidential information recording, and logic data processing^3–5^. Last decades have witnessed the exponential advances of silicon-based nonvolatile optoelectronic memories^5–7^. However, the continued device miniaturization and the feasibility of integration into flexible, wearable, and transparent circuits greatly restrict the development of conventional silicon-based optoelectronic memories^8,9^.

Two-dimensional (2D) thin layered materials have been considered as promising building blocks for the next-generation electronic and optoelectronic devices due to their extraordinary and unique properties^10,11^. The 2D thin layered structure enables their immunity against the short channel effects^9,12^, while the mechanical strength and structural flatness allow their integration into flexible and wearable circuits^13,14^. In comparison to the massive research of 2D materials-based photoconductors and photodiodes^15–19^, the nonvolatile optoelectronic memories fabricated by these materials are rarely investigated. Mechanically exfoliated few-layer copper indium selenide (CuIn_7_Se_11_) was firstly applied in 2D thin layered optoelectronic memory^20^, however, the short retention time (approximately 50 s) and low current switching ratio (less than 10) hindered its application in image sensing. Monolayer molybdenum disulfide (MoS_2_) optoelectronic memory was reported to possess long retention time (approximately 10^4^ s), while suffering from moderate switching ratio (approximately 4700) and limited data storage capacity (8 storage levels)^21^. Besides the memory devices fabricated by single 2D crystals, graphene/MoS_2_ vertical heterostructure was also realized with low switching ratio (less than two)^22^. Recently, the 2D materials-based semifloating-gate field-effect-transistor and metallic gold nanoparticles/crosslinked poly(4-vinylphenol)/MoS_2_ heterostructure memories have been reported, demonstrating high switching ratio, which are promising candidates for thin layered multibit optoelectronic memory^23–25^. In order to enhance the data storage capability of a single optoelectronic memory, it is essential to increase the number of storage level, which is typically reflected by the difference of reading currents between a programmed state and an erased state^1^.

In addition to data storage capability, an optoelectronic memory that can distinguish light wavelength is highly demanded for color sensing in digital imaging^26–28^. Spectrum distinction in the commercial color sensors is achieved by combining the broadband inorganic semiconductor-based photodetectors with a set of optical filters, including organic dye filters and plasmonic color filters^28–30^. However, these filters not only increase the architectural complexity and cost of the color sensors, but also limit the pixel density in imaging system^31,32^. Furthermore, the image sharpness and color constancy would be degraded by the interference effect of the optical filter^31^. In order to simplify system structure, reduce fabrication cost, and improve image quality, it is essential to design filter-free optoelectronic memory in a single device with the same spectrum distinguishing capability as human eyes^33^.

Here, we report a multibit nonvolatile optoelectronic memory-based on a hybrid structure of thin layered tungsten diselenide (WSe_2_) and boron nitride (BN). The storage current of the WSe_2_/BN optoelectronic memory can be effectively modulated by backgate, resulting in a memory switching ratio approximately 1.1 × 10^6^. This large switching ratio coupled with the optically tunable characteristic ensures over 128 distinct storage levels (7 bit storage). The device is also highly reliable, as reflected by its long retention time and large number of program-erase testing cycles. Moreover, the wavelength distinguishing property of the memory promises WSe_2_/BN heterostructure for the application in filter-free color image sensor. This is illustrated by fabricating an array of the heterostructure-based optoelectronic memory on large area chemical vapor deposition (CVD) WSe_2_, in which a specific image recording the three primary lights (red, green, and blue) is created. The optoelectronic memory based on black phosphorous (BP) and BN heterostructure is also demonstrated with excellent data storage property.

## Results

### Operational mechanism of WSe_2_/BN optoelectronic memory

**Fig. 1:**
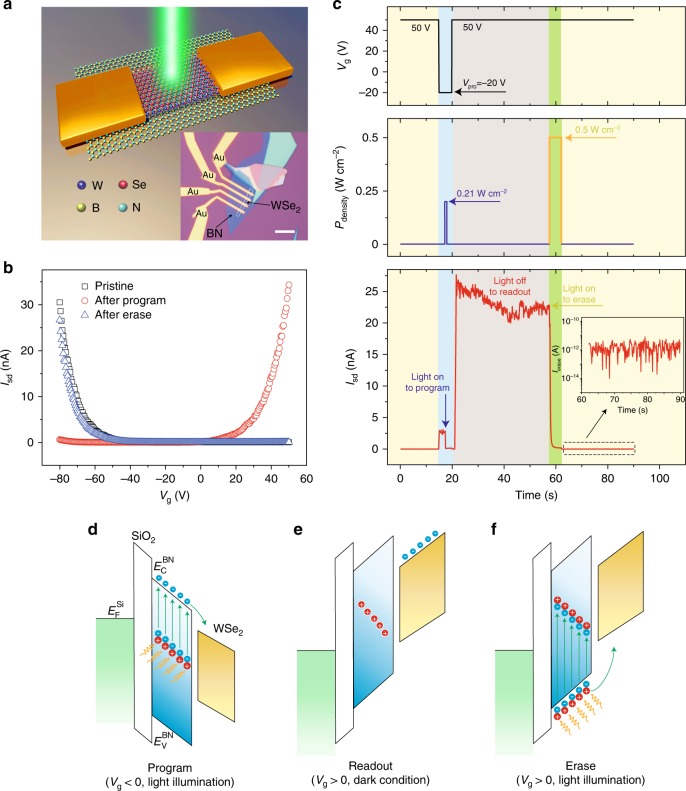
Operational mechanism of the WSe_2_/BN heterostructure-based optoelectronic memory. **a** Schematic illustration of the optoelectronic memory fabricated by transferring WSe_2_ flake on BN flake. The dark blue, red, yellow, and light blue balls represent the W, Se, B, and N atoms, respectively. Inset: optical image of the fabricated WSe_2_/BN heterostructure. The scale bar is 10 µm. **b** The transfer curves of pristine, programmed, and erased WSe_2_/BN device at *V*_sd_ = 1 V. The programming gate is −20 V. The transport characteristic of the WSe_2_/BN FET was converted from hole-domination to electron-domination after programming, followed by a return to hole-domination after erasing. **c** A single program-readout-erase cycle of the WSe_2_/BN memory at *V*_pro_ = −20 V. The shaded backgrounds in light yellow, blue, gray, and green represent the device states as pristine, programming, readout, and erasing, respectively. Inset: plot of the erased current. Schematic of the band diagrams of the WSe_2_/BN optoelectronic memory under programming (**d**), readout (**e**), and erasing processes (**f**). The rectangle in grayish green represents the Si band diagram, while the parallelograms in white, blue and khaki represent the band diagrams of SiO_2_, BN, and WSe_2_, respectively. The red and blue circles represent the positive charges and electrons, respectively. ${E}_{\mathrm{F}}^{\mathrm{Si}}$ is the Fermi level energy of Si substrate. ${E}_{\mathrm{C}}^{\mathrm{BN}}$ and ${E}_{\mathrm{V}}^{\mathrm{BN}}$represent the minimum energy of conduction band and the maximum energy of valance band of BN, respectively

Figure 1a shows the schematic of the hybrid WSe_2_/BN optoelectronic memory fabricated in a field-effect-transistor (FET) structure, in which monolayer WSe_2_ flake is transferred on top of a BN flake. The crystallinity and thicknesses of both WSe_2_ and BN are characterized by Raman and AFM, respectively (Supplementary Figs. 1 and 2 and Supplementary Note 1). The hybrid WSe_2_/BN FET demonstrates typical p-type transport behavior (Fig. 1b), in good agreement with previous reports^34^.

Figure 1c shows the dynamic behavior of the WSe_2_/BN optoelectronic memory in a single cycle, which includes programming, readout, and erasing processes. The mechanism of these three processes is illustrated in Fig. 1d–f, respectively. Since WSe_2_ is intrinsically p-type, the current of the pristine WSe_2_/BN FET in the positive gate regime is considerably low. In order to program the memory, the device is illuminated by a light pulse (duration 0.5 s, wavelength 405 nm, intensity 210 mW cm^−2^) under negative gate pulse, which results in remarkable excitation of electrons from the mid-gap donor-like states (defects) of BN to its conduction band (Fig. 1d)^35^. The photon-excited electrons in BN conduction band can transfer into WSe_2_ driven by the electric field, leaving the positive charges localized in middle of the BN bandgap. It is worth noting that these localized positive charges in BN can effectively screen the negative gate and hence weaken the electric field exerting on WSe_2_ during the programming process (Supplementary Fig. 3). The elimination of the effective electric field in BN symbolizes the termination of the programming process. The positive charges can be stored in BN even after removing the negative gate and switching off the light, thereby serving as an effective local gate and generating a stable electron-storage effect in WSe_2_. The storage current *I*_store_ after programming is readout at a positive gate under dark condition, as shown in Fig. 1e. When the gate was switched to 50 V, a sharp rise of current was observed, followed by stabilization at around 22 nA, which indicates the nonvolatile property of the WSe_2_/BN memory (Fig. 1c).

The erasing operation is realized by applying positive gate on WSe_2_/BN with light illumination (Fig. 1f). In this process, the ionized positive defects in BN are filled by photon-excited electrons from BN valence band, generating large quantity of holes. Attributing to the external electric field, the generated holes in BN move to WSe_2_. As a consequence, the localized positive charges in BN are vanished and the device returns to its original hole-domination transport behavior after erasing, as shown in Fig. 1b. It is noted that the charge erasing is completed in 2 s, indicating the fast switching of the WSe_2_/BN memory. Moreover, the average erased current *I*_erase_ is read as 1.3 × 10^−12^ A (inset of Fig. 1c), contributing to a large switching ratio with *I*_store_/*I*_erase_ approximately 1.7 × 10^4^.

### Programming gate controlled optoelectronic memory

**Fig. 2:**
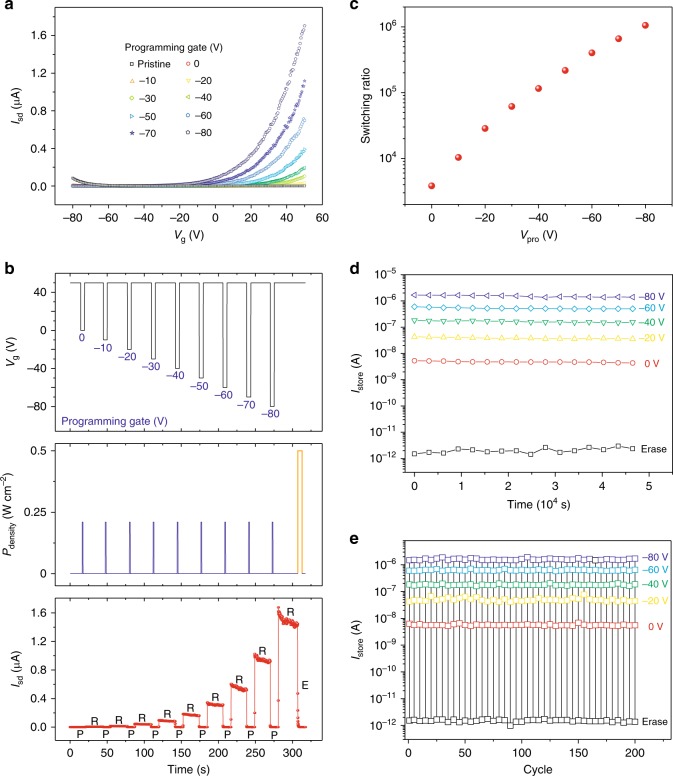
Programming gate controlled WSe_2_/BN optoelectronic memory and reliability tests. **a** Transfer characteristics evolution of the memory device with respect to *V*_pro_. **b** Dynamic behavior of the memory at different programming gate ranging from 0 to −80 V with step −10 V. The readout current increases stepwise, generating nine clear storage states. **c** The plot of switching ratio as a function of *V*_pro_. Current retention (**d**) and cycling tests (**e**) for the WSe_2_/BN memory at different *V*_pro_ (*V*_pro_ = 0, −20, −40, −60, and −80 V). The squares in black represent the device in the erased state, while the symbols in red, yellow, green, blue, and purple represent the programmed state under *V*_pro_ = 0, −20, −40, −60, and −80 V, respectively

Figure 2a shows the transfer characteristics evolution of the WSe_2_/BN device under different programming gate (*V*_pro_ from 0 V to −80 V). The on current in electron-domination regime gradually rises with increasing *V*_pro_, which indicates a significant gate-tunable electron-doping effect on WSe_2_. The dynamic behavior of the WSe_2_/BN optoelectronic memory modulated by *V*_pro_ is also investigated (Fig. 2b). When the *V*_pro_ is switched from 0 V to −80 V with −10 V step, the storage current increases stepwise, generating 9 clear storage states. The switching ratio at different *V*_pro_ is obtained through extracting the storage current. As shown in Fig. 2c, the switching ratio is largely enhanced from 3.8 × 10^3^ to 1.1 × 10^6^ when *V*_pro_ increases from 0 to −80 V. The larger negative backgate can better stabilize the generated positive charges in middle of the BN bandgap and facilitate the formation of higher concentration of positive charges in BN, which can result in more effective electron-doping and greater storage current in WSe_2_.

In order to evaluate the reliability of our optoelectronic memory for practical application, we investigate both the retention time and the cyclic program/erase endurance of our device. Figure 2d shows the nonvolatile and data retention property of the memory under different *V*_pro_, in which highly stabilized storage states are observed within the time range of 4.5 × 10^4^ s. It is worth noting that the memory is kept isolated in the absence of any external perturbation (no voltage and no light) after programming and the storage currents in Fig. 2d were extracted in a fixed interval of 3.1 × 10^3^ s (Supplementary Fig. 4). The retention curves are then extrapolated to 10 years, which is a technical requirement for commercial nonvolatile memory (Supplementary Fig. 5). Nearly half the stored currents are expected to be maintained after 10 years with clearly distinguished storage states, indicating the excellent data retention property of the WSe_2_/BN optoelectronic memory. Figure 2e displays the repeatability of the program/erase process at different *V*_pro_ (0, −20, −40, −60, and −80 V) for 200 cycles. The deviation from the average value of the readout currents for each *V*_pro_ is less than 10%, indicating that the programmed data is highly reproducible. Given the ultralong retention time and robust cyclic endurance, WSe_2_/BN optoelectronic memory demonstrates potential for practical applications.

### One hundred thirty current-level optoelectronic memory

**Fig. 3:**
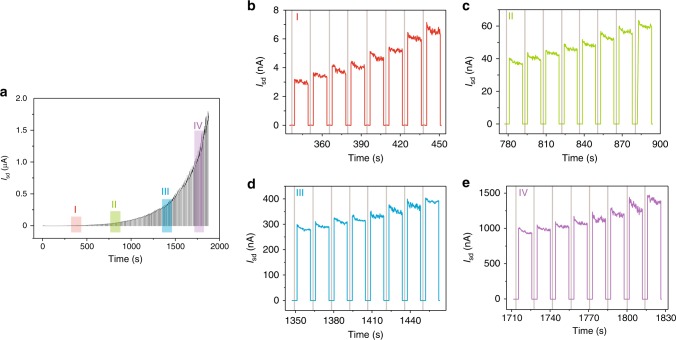
One hundred thirty storage states. **a** The dynamic behavior of the memory under the exposure of light pulse (*t*_pro_ = 0.5 s, *λ* = 405 nm, *P* = 2 nW) at *V*_pro_ = −80 V, and the enlargements in region I (**b**), II (**c**), III (**d**), and IV (**e**), respectively. The regions shaded in red, green, blue, and purple in **a** correspond to the enlarged regions plotted in the same colors in **b**–**e**, respectively

WSe_2_/BN optoelectronic memory demonstrates high switching ratio, which indicates the possibility to achieve multibit memory with excellent storage capability. Figure 3a shows the dynamic behavior of the memory under periodic exposures of light pulse (130 pulses, *t*_pro_ = 0.5 s, *λ* = 405 nm, *P* = 2 nW) at *V*_pro_ = −80 V, and Fig. 3b–e are the enlarged regions I–IV, respectively. The storage current rises progressively with increasing the pulse number, a phenomenon represents the continual accumulation of electrons in WSe_2_ as prolonging the light exposure on the memory. One hundred thirty light pulses are employed in our experiment, resulting in 130 effective storage states before the current saturation (Supplementary Fig. 6 and Supplementary Note 2). The reliability of the storage states is evaluated by comparing the gaps of two neighboring states and their noise (Supplementary Fig. 7 and Supplementary Note 3). The result demonstrates that all the storage states for our WSe_2_/BN optoelectronic memory are valid. We have repeated the program-erase cycle for 20 times, and at least 130 valid storage levels were achieved for all the 20 independent cycles, which suggests the excellent repeatability of the WSe_2_/BN memory. The dynamic behavior of the 1st, 10th, and 20th cycle is shown in Supplementary Fig. 8 for illustration. The storage current rises progressively with increasing pulses, followed by a gradual saturation when the pulse number goes beyond 130, which is consistent with the result shown in Fig. 3a. The *Y* ratios for all the selected cycles have also been plotted in Supplementary Fig. 8, indicating the validity of the 130 states for each cycle by ensuring *Y* > 1. We propose that the repeatability is mainly due to the similar erased currents (in the magnitude of 10^−12^ A) after each cycle (Supplementary Fig. 9). The same level of base current means the same starting point for each independent cycle, which ensures the high repeatability of the WSe_2_/BN memory. The weak fluctuation of the storage currents can be attributed to the noise and the instability of our laser pulse system. Therefore, we have successfully fabricated a 130 current-level optoelectronic memory by using 2D thin layered heterostructure with data storage capacity over 7 bit (128 levels). It is worth mentioning that the number of storage states in our memory is limited by the noise level. More storage states could be achieved by further minimizing the noise. Moreover, the memory device can be operated under the light energy of 1 nJ (light power multiplies exposure time), indicating its high light sensitivity.

### Spectrum distinction of WSe_2_/BN optoelectronic memory

**Fig. 4:**
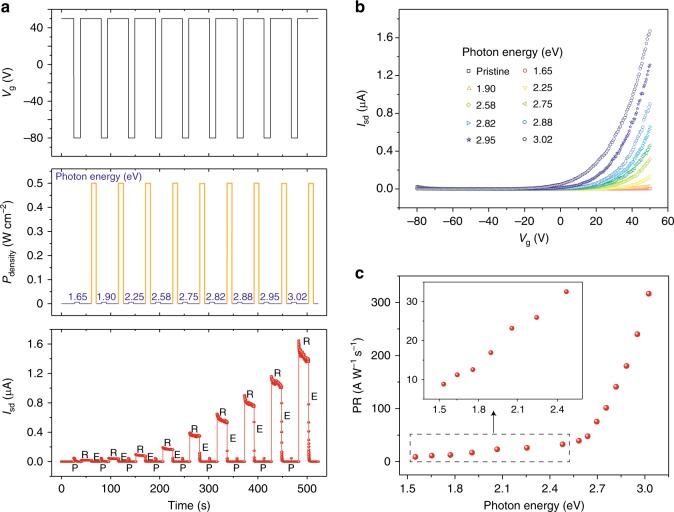
Wavelength distinguishing ability of WSe_2_/BN optoelectronic memory. **a** Dynamic behavior of the WSe_2_/BN memory illuminated by a wide spectrum of lights with wavelength ranging from 750 to 410 nm. Nine distinguishable storage states are clearly observed under different programming wavelengths. **b** The corresponding transfer characteristic with respect to wavelength after each programming. **c** Programming rate as a function of photon energy. Inset: plot of the enlargement from 1.55 to 2.48 eV

Optoelectronic memory with the capability of wavelength discrimination is particularly superior for the application of filter-free color image sensor. Figure 4a shows the dynamic behavior of the WSe_2_/BN optoelectronic memory illuminated by light with wavelengths from 750 (1.65 eV) to 410 nm (3.02 eV). The corresponding readout current increases stepwise from 3 × 10^−2^ to 1.5 μA when the wavelength decreases from 750 to 410 nm. The on current in the electron-domination regime after each programming process also rises gradually when shortening the programming wavelength (Fig. 4b), in good agreement with the dynamic results. Moreover, the storage states at different wavelengths are highly distinct, indicating excellent wavelength distinguishing capability of the WSe_2_/BN optoelectronic memory. In order to quantify the modulating ability of different wavelengths, we define the programming rate (PR) as below,

1 $${\mathrm{PR}} = \frac{{I_{{\mathrm{store}}}}}{{P_{{\mathrm{density}}} \cdot S \cdot t_{{\mathrm{pro}}}}}$$

Where *P*_density_ is the power density of light, *S* is the device area. The relationship between PR and photon energy is plotted in Fig. 4c. The monotonic increase of PR with photon energy suggests that light with higher photon energy (shorter wavelength) can induce greater amount of stable localized positive charges in BN in a unit time. It is to note that the PR starts increasing rapidly when the photon energy exceeds 2.6 eV. Previous works have reported that the light absorption by the donor-like states in BN is strongly enhanced at the photon energy around 2.6 eV, originating from the nitrogen vacancy in BN crystal^35–37^. This phenomenon is in good agreement with our experimental result.

### Integrated memory matrix for color image sensor

**Fig. 5:**
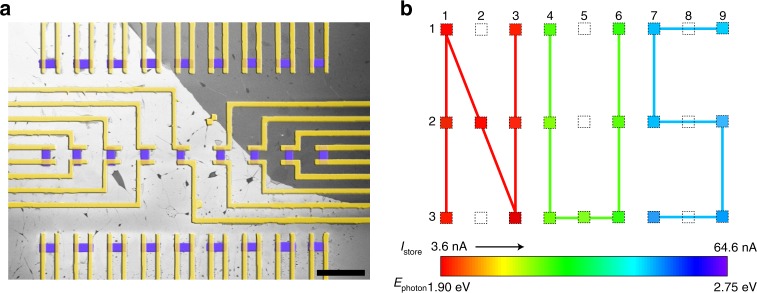
Integrated WSe_2_/BN pixel matrix for color image sensor. **a** False-colored SEM image of the fabricated WSe_2_/BN pixel matrix in arrays (three rows and nine columns). The scale bar is 10 µm. The channel length is almost the same for each pixel after deep RIE etching, approximately 2 µm. **b** The corresponding schematic of the pixel matrix with selective exposure under three different wavelength lights (red 638 nm, green 515 nm, and blue 473 nm). The programming gate, time and laser power were fixed the same as −80 V, 2 s, and 10 nW, respectively for each exposure. The color bar was achieved by reading the storage current of pixel 11 under different lights with photon energy (*E*_photon_) from 1.90 to 2.75 eV

WSe_2_/BN optoelectronic memory are capable of detecting and discriminating lights with different wavelengths, indicating its potential for the application of filter-free color image sensors. Besides, in order to realize practical application, it is essential to fabricate large quantity of image sensors in an integrated circuit. The large area CVD grown WSe_2_ is used to fabricate the sensor matrix, with its monolayer characteristic confirmed by the Raman and PL spectra (Supplementary Fig. 10). Figure 5a displays a false-colored SEM image of the integrated pixel matrix with 27 WSe_2_/BN image sensors arranged in a 3 × 9 array. The darker color in the upper right part of the image is due to the slight thickness variation of BN substrate (Supplementary Fig. 11). It is worth noting that each pixel with channel length approximately 2 µm is able to function independently since they are isolated by e-beam lithography (EBL) and deep reactive ion etch (RIE). In order to investigate the color sensing property of an individual pixel and the image capture ability of the matrix, three laser beams (spot diameter 3 μm) with different wavelengths (red 638 nm, green 515 nm, and blue 473 nm) are used to expose the selected pixels in sequence. Three pixel groups (group I: 11, 13, 21, 22, 23, 31, 33; group II: 14, 16, 24, 26, 34, 35, 36; and group III: 17, 19, 27, 29, 37, 39) record the three different lights, while the other pixels are left unexposed. Figure 5b demonstrates the corresponding schematic of the matrix after selective exposures. The image NUS is captured in the matrix, in which N, U, and S record the three different lights (red, green, and blue), respectively. More intriguingly, the three different pixel groups display three distinct storage states with slightly fluctuated *I*_store_ 5, 12, and 31 nA, respectively (Supplementary Fig. 12), which enables the realization of a color image.

### BP/BN optoelectronic memory

The heterostructure-based optoelectronic memory can be applied to other 2D crystals. BP, a 2D material, has been widely investigated recently due to its superior optical and electrical transport properties^17,38,39^. The BP/BN optoelectronic memory fabricated in the same configuration also demonstrates excellent data storage ability (Supplementary Figs. 13–15 and Supplementary Note 4). The storage states of the BP/BN memory can be effectively modulated by light wavelength and the device possesses high reliability (Supplementary Figs. 16 and 17), which are similar to the WSe_2_/BN memory. However, the switching ratio of the BP/BN memory (around 415) is lower than that of the WSe_2_/BN device, which is mainly due to the large off current of our BP FET. It is possible to further improve the switching ratio by selecting thinner BP flakes^38^. Unexpectedly, the BP/BN heterostructure is particularly sensitive to weak light. An ultrahigh photo responsivity approximately 1.2 × 10^7^ A W^−1^ is observed for the device, making BP/BN heterostructure a promising candidate for photodetector application (Supplementary Fig. 18 and Supplementary Note 5).

## Discussion

We have demonstrated a multibit nonvolatile optoelectronic memory-based on the 2D thin layered WSe_2_/BN heterostructure. The programming and erasing processes of the memory are controlled by tuning the polarity of the backgate. During the programming process, the photon-generated electrons from the mid-gap donor-like states of BN transfer into WSe_2_ under negative gate, and lead to the storage of localized positive charges in middle of the BN bandgap, which can serve as effective local gate to modulate the transport behavior of WSe_2_. During the erasing process, by applying positive gate, the localized positive charges stored in BN recombine with the photo-excited electrons from the BN valence band, thereby eliminating the local gating effect and restoring the original transport behavior of WSe_2_. The switching ratio of the memory can reach up to 1.1 × 10^6^ under *V*_pro_ = −80 V, ensuring 130 distinguishable storage states. The memory device exhibits excellent performance for data retention (over 4.5 × 10^4^ s) and cyclic endurance (exceeding 200 cycles). Moreover, the WSe_2_/BN device is able to discriminate wavelengths in the full visible spectrum, indicating its potential to be used as filter-free color image sensor. This concept is further supported by the realization of WSe_2_/BN pixel matrix which captures a specific image recording the three primary colors. The discovery of 2D thin layered heterostructure-based optoelectronic memory provides a simple method to achieve multibit memory device. The realization of pixel matrix indicates the possibility of fabricating 2D thin layered image sensors in integrated circuit, which paves the way for the next-generation optoelectronic memories.

## Methods

### Fabrication and characterization of optoelectronic memory

The hybrid structure of WSe_2_ and BN was achieved by a dry transfer method^40^. Firstly, few-layer BN flakes with thickness around 10 nm were mechanically exfoliated onto 300 nm SiO_2_/Si substrate. In the following, the WSe_2_ flake exfoliated on a transparent polydimethylsiloxane (PDMS) substrate was aligned on the BN flake using optical microscope. After the alignment, the PDMS film was pressed on the Si substrate for 2 min followed by a slow lift up, during which the WSe_2_ flake was transferred onto the BN flake. The BP/BN heterostructure was obtained by the same dry transfer method. To avoid oxidation of the BP flakes, the experiment was carried out in a glovebox. Standard EBL was employed to define the memory channel and the electrodes (Ti/Au) was deposited by thermal evaporation. After lift-off, the memory device was loaded into a vacuum chamber (pressure below 10^−7^ mbar) for characterizations. The optoelectronic measurements were conducted by using an Agilent 2912A source measure unit. Four laser beams (638, 515, 473, and 405 nm) and an exon light source configured with a monochromator were used to program or erase the memories. The light density was calibrated by THORLABS GmbH (PM 100A) power meter.

### CVD WSe_2_ growth

A one-zone tube furnace was used to grow WSe_2_^41^. Hundred milligram Se powder (Sigma-Aldrich, 99.5%) was loaded at upstream, and kept at 300 °C during growth. A mixture of WO_2.9_ (30 mg, Alfa Aesar, 99.99%) and NaCl (10 mg, Sigma-Aldrich, 99.5%) was loaded at the center of reaction zone. The temperature of reaction zone gradually increased to 830 °C in 22 min, and cooled down to room temperature after staying at 830 °C for 15 min. Pure Ar and H_2_ (90/10 sccm) were used as carrying gas.

### Fabrication of 2D pixel matrix

CVD WSe_2_ was transferred onto BN flake by a wet transfer method^42^. Firstly, the as-grown WSe_2_ on Si substrate coated with 300 nm SiO_2_ was spin coated by polymethyl methacrylate (PMMA). The Si substrate was then left in 2 M KOH solution for several hours, yielding PMMA coated WSe_2_ film. The WSe_2_/PMMA film was washed in deionized water for three times before transferring onto exfoliated BN flake. EBL was used to pattern pixel matrix on the large area heterostructure followed by RIE to isolate each pixel. The electrodes were then patterned using standard EBL, thermal deposition, and lift-off.

### Data availability

The data that supports the plots within this paper and other findings of this study are available from the corresponding authors upon reasonable request.

## Electronic supplementary material

Supplementary Information

## References

[1] Leydecker T (2016). Flexible nonvolatile optical memory thin-film transistor device with over 256 distinct levels based on an organic bicomponent blend. Nat. Nano.

[2] Star A, Lu Y, Bradley K, Grüner G (2004). Nanotube optoelectronic memory devices. Nano Lett..

[3] Sun C (2015). Single-chip microprocessor that communicates directly using light. Nature.

[4] Xu Q, Schmidt B, Pradhan S, Lipson M (2005). Micrometre-scale silicon electro-optic modulator. Nature.

[5] Fossum ER (1997). CMOS image sensors: Electronic camera-on-a-chip. IEEE Trans. Electron Dev..

[6] Shacham A, Bergman K, Carloni LP (2008). Photonic networks-on-chip for future generations of chip multiprocessors. IEEE Trans. Comput..

[7] Batten C (2009). Building many-core processor-to-DRAM networks with monolithic CMOS silicon photonics. IEEE Micro.

[8] Venema L (2011). Silicon electronics and beyond. Nature.

[9] Schwierz F (2010). Graphene transistors. Nat. Nano.

[10] Neto AC, Guinea F, Peres NM, Novoselov KS, Geim AK (2009). The electronic properties of graphene. Rev. Mod. Phys..

[11] Wang QH, Kalantar-Zadeh K, Kis A, Coleman JN, Strano MS (2012). Electronics and optoelectronics of two-dimensional transition metal dichalcogenides. Nat. Nano.

[12] Yoon Y, Ganapathi K, Salahuddin S (2011). How good can monolayer MoS_2_ transistors be?. Nano Lett..

[13] Kim KS (2009). Large-scale pattern growth of graphene films for stretchable transparent electrodes. Nature.

[14] Georgiou T (2013). Vertical field-effect transistor based on graphene-WS_2_ heterostructures for flexible and transparent electronics. Nat. Nano.

[15] Xia F, Mueller T, Lin Ym, Valdes-Garcia A, Avouris P (2009). Ultrafast graphene photodetector. Nat. Nano.

[16] Lopez-Sanchez O, Lembke D, Kayci M, Radenovic A, Kis A (2013). Ultrasensitive photodetectors based on monolayer MoS_2_. Nat. Nano.

[17] Xiang D (2015). Surface transfer doping induced effective modulation on ambipolar characteristics of few-layer black phosphorus. Nat. Commun..

[18] Baugher BW, Churchill HO, Yang Y, Jarillo-Herrero P (2014). Optoelectronic devices based on electrically tunable p–n diodes in a monolayer dichalcogenide. Nat. Nano.

[19] Ross JS (2014). Electrically tunable excitonic light-emitting diodes based on monolayer WSe_2_ pn junctions. Nat. Nano.

[20] Lei S (2014). Optoelectronic memory using two-dimensional materials. Nano Lett..

[21] Lee J (2017). Monolayer optical memory cells based on artificial trap-mediated charge storage and release. Nat. Commun..

[22] Roy K (2013). Graphene-MoS_2_ hybrid structures for multifunctional photoresponsive memory devices. Nat. Nano.

[23] Li D (2017). Two-dimensional nonvolatile programmable p–n junctions. Nat. Nano.

[24] Li D, Chen M, Zong Q, Zhang Z (2017). Floating-gate manipulated graphene-black phosphorus heterojunction for nonvolatile ambipolar schottky junction memories, memory inverter circuits, and logic rectifiers. Nano. Lett..

[25] Lee D (2016). Multibit MoS_2_ photoelectronic memory with ultrahigh sensitivity. Adv. Mater..

[26] Goossens S (2017). Broadband image sensor array based on graphene–CMOS integration. Nat. Photon..

[27] Park H (2014). Filter-free image sensor pixels comprising silicon nanowires with selective color absorption. Nano Lett..

[28] Jansen van Vuuren RD, Armin A, Pandey AK, Burn PL, Meredith P (2016). Organic photodiodes: the future of full color detection and image sensing. Adv. Mater..

[29] Burgos SP, Yokogawa S, Atwater HA (2013). Color imaging via nearest neighbor hole coupling in plasmonic color filters integrated onto a complementary metal-oxide semiconductor image sensor. ACS Nano.

[30] Yokogawa S, Burgos SP, Atwater HA (2012). Plasmonic color filters for CMOS image sensor applications. Nano Lett..

[31] Lin Q, Armin A, Burn PL, Meredith P (2015). Filterless narrowband visible photodetectors. Nat. Photon..

[32] Gao L (2017). Flexible filter‐free narrowband photodetector with high gain and customized responsive spectrum. Adv. Funct. Mater..

[33] Gao L (2016). Broadband, sensitive and spectrally distinctive SnS_2_ nanosheet/PbS colloidal quantum dot hybrid photodetector. Light Sci. Appl..

[34] Fang H (2012). High-performance single layered WSe_2_ p-FETs with chemically doped contacts. Nano Lett..

[35] Ju L (2014). Photoinduced doping in heterostructures of graphene and boron nitride. Nat. Nano.

[36] Taniguchi T, Watanabe K (2007). Synthesis of high-purity boron nitride single crystals under high pressure by using Ba–BN solvent. J. Cryst. Growth.

[37] Jaffrennou P (2007). Origin of the excitonic recombinations in hexagonal boron nitride by spatially resolved cathodoluminescence spectroscopy. J. Appl. Phys..

[38] Li L (2014). Black phosphorus field-effect transistors. Nat. Nano..

[39] Xia F, Wang H, Jia Y (2014). Rediscovering black phosphorus as an anisotropic layered material for optoelectronics and electronics. Nat. Commun..

[40] Castellanos-Gomez A (2014). Deterministic transfer of two-dimensional materials by all-dry viscoelastic stamping. 2D Mater..

[41] Li S (2015). Halide-assisted atmospheric pressure growth of large WSe_2_ and WS_2_ monolayer crystals. Appl. Mater. Today.

[42] Li X (2009). Large area synthesis of high-quality and uniform graphene films on copper foils. Science.

