# Supplementary material for: Two-dimensional multibit optoelectronic memory with broadband spectrum distinction
Source: Nat Commun. 2018 Jul 27;9:2966. doi: 10.1038/s41467-018-05397-w (PMC6063921; doi:10.1038/s41467-018-05397-w)
Supplement: Supplementary file 1 — Supplementary Information [file 41467_2018_5397_MOESM1_ESM.pdf]

Supplementary Information

Two Dimensional Multi-bit Optoelectronic Memory with Broadband Spectrum Distinction

Xiang et al.

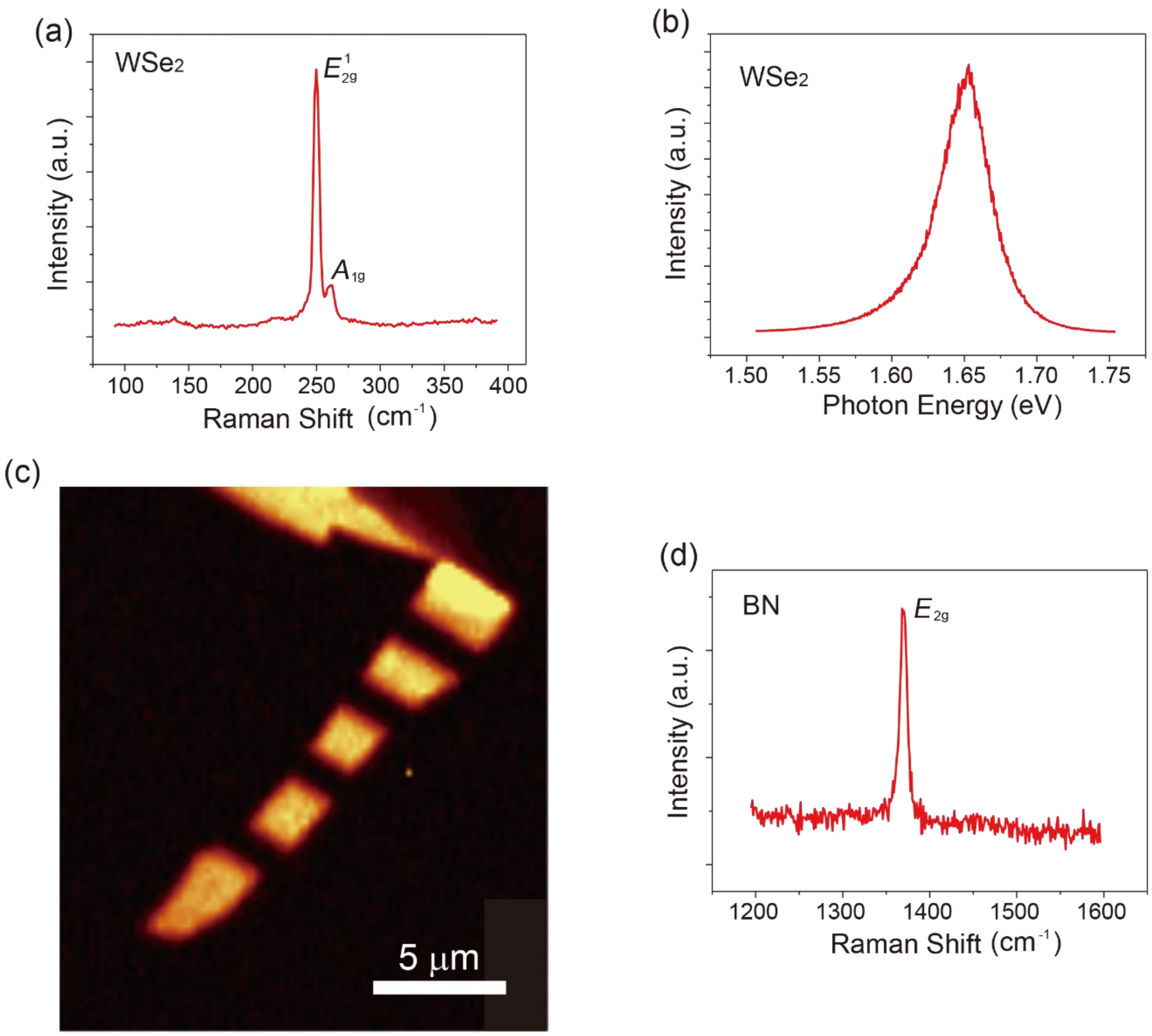

Supplementary Figure 1 | Raman and photoluminescence (PL) spectra of WSe_2 and BN crystals. (a) Raman and PL **(b)** spectra of the exfoliated WSe_2 flake. **(c)** Raman intensity map of the E^1_{2g} mode of the WSe_2 flake. **(d)** Raman spectrum of the exfoliated BN flake.

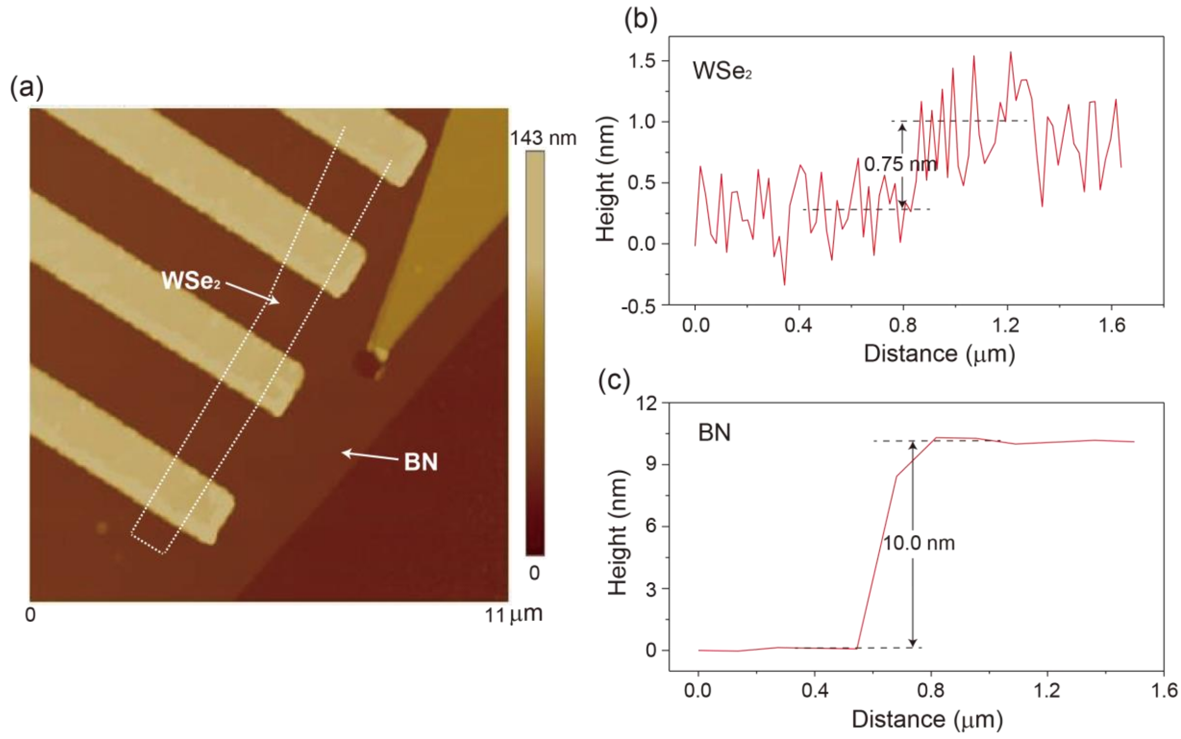

Supplementary Figure 2 | AFM images of WSe_2 and BN flakes. (a) AFM image of the as-fabricated WSe_2/BN device. Line profiles at the edges of the WSe_2 **(b)** and BN **(c)** flakes. The thickness of WSe_2 flake is around 0.75 nm (monolayer), in good agreement with the Raman result. BN flake shows a thickness of 10 nm, corresponding to layer number of 20.

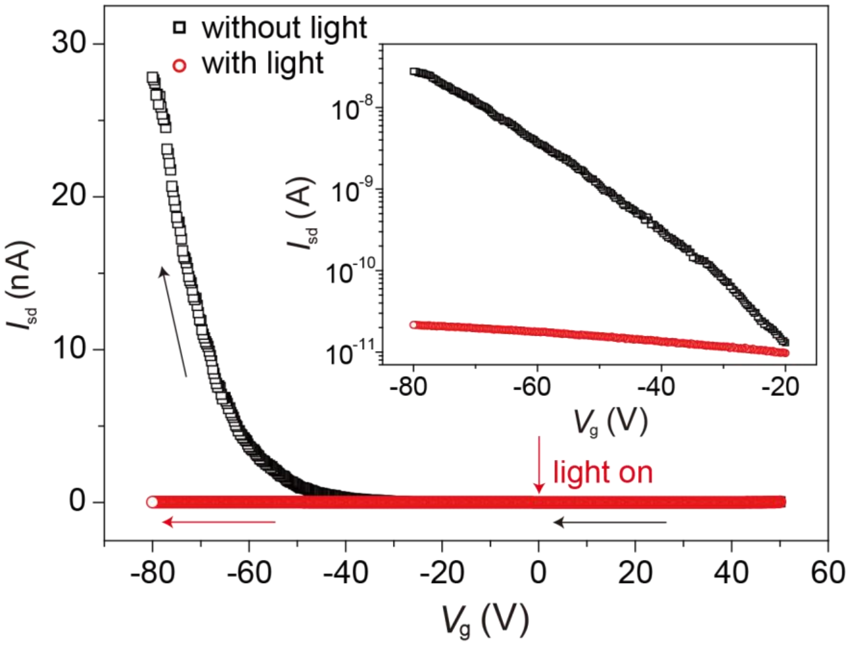

Supplementary Figure 3 | Backgate screening effect of WSe_2/BN device under light illumination. Backward transfer characteristics (V_g from 50 V to -80 V) of the WSe_2/BN device under dark and light conditions. The light is switched on when V_g is sweeping from 0 V to -80 V. The on current of the pristine WSe_2/BN device at negative gate regime reaches up to 28 nA, exhibiting a typical p-type transport behavior. In contrast, when the light is on, the hole transport current drops dramatically. This phenomenon is mainly due to the generation of ionized positive defects in BN under light illumination[1]. These positively charged defects are able to effectively screen the electric field induced by the negative backgate exerting on the WSe_2, which, in turn, resulting in the significant decrease of the hole transport current in the WSe_2 device.

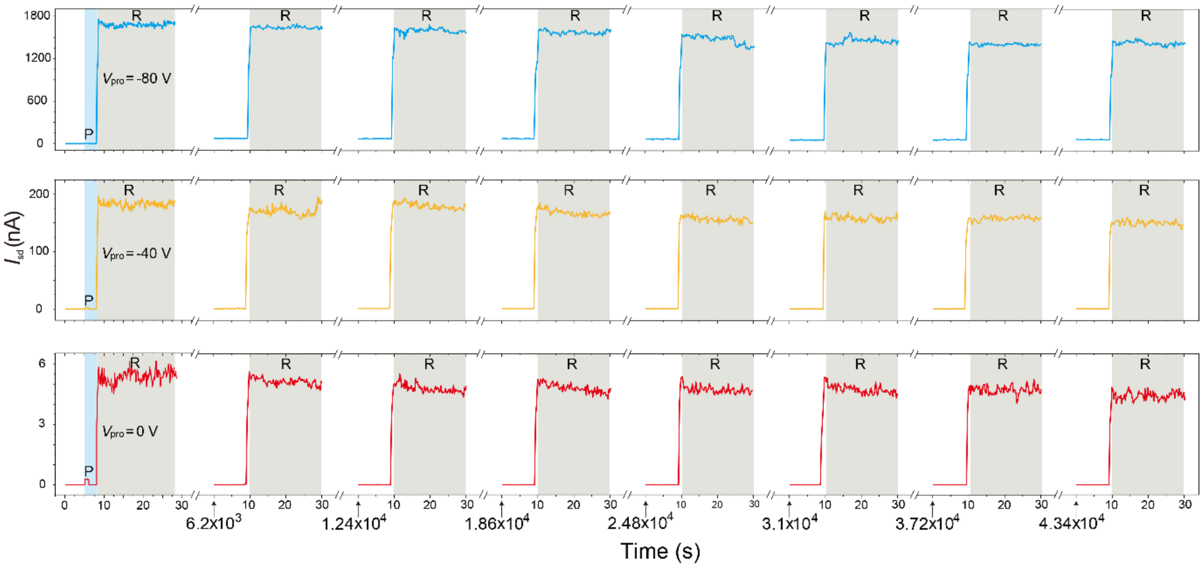

Supplementary Figure 4 | Non-volatile property of the WSe_2/BN memory. Readout current as a function of waiting time up to 4.34x10^4 s. P and R refer to program and readout respectively. The graph was spread separately with an interval of 6.2x10^3 s. In order to further investigate the non-volatility of our memory, we analyzed its charge retention time. The memory was kept in the absence of external perturbation (no voltage and no light) after programming, and the storage currents were readout in a fixed interval (3.1×10^3 s). Supplementary Fig.4 shows the storage currents as a function of waiting time up to 4.34x10^4 s at three different programming gates (V_{pro} = 0 V, -40 V, -80 V). To simplify the graph, we plotted the data with an interval of 6.2x10^3 s. The storage currents almost keep unchanged in the time range 4.34x10^4 s for all the programming gates, which verifies the memory is retained in the absence of external perturbation.

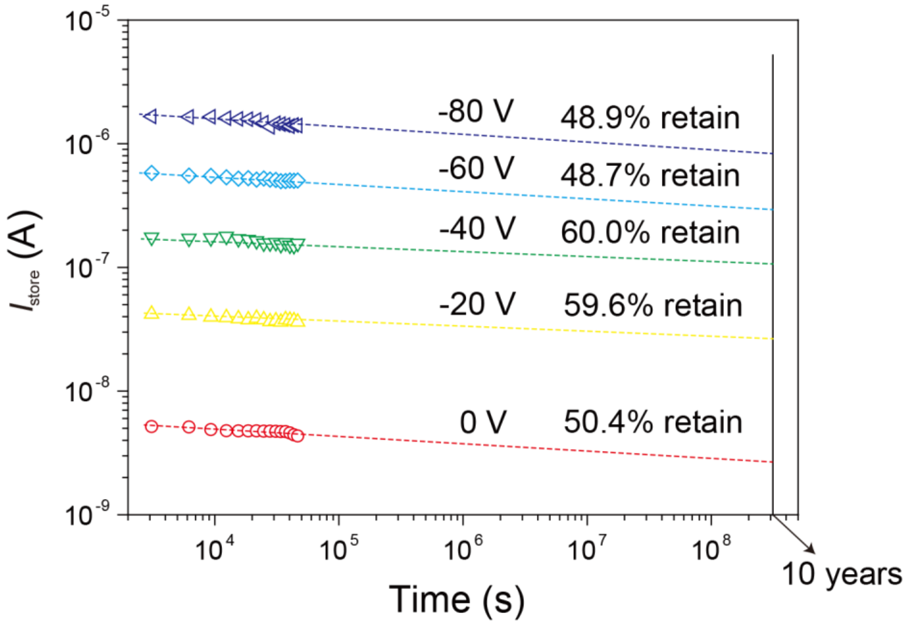

Supplementary Figure 5 | 10 years charge retention. 10 years linear extrapolation of the storage currents without electrical and optical pulses after programming at different programming gates (V_{pro} = 0 V, -20 V, -40 V, -60 V, and -80 V). Nearly half the storage currents can be retained after 10 years for all V_{pro}, which indicates the practical application of the WSe_2/BN optoelectronic memory.

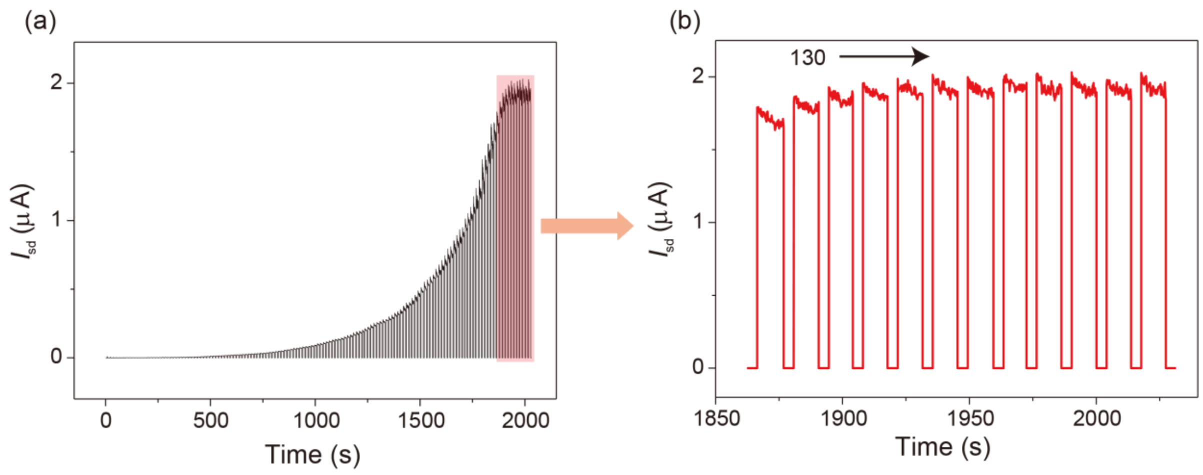

Supplementary Figure 6 | Saturation of the storage current. (a) Dynamic behavior of the memory under the exposure of light pulse (t_{pro} = 0.5 s, λ = 405 nm, P = 2 nW) at V_{pro} = -80. **(b)** Enlargement of the dynamic behavior from 130th storage level onwards.

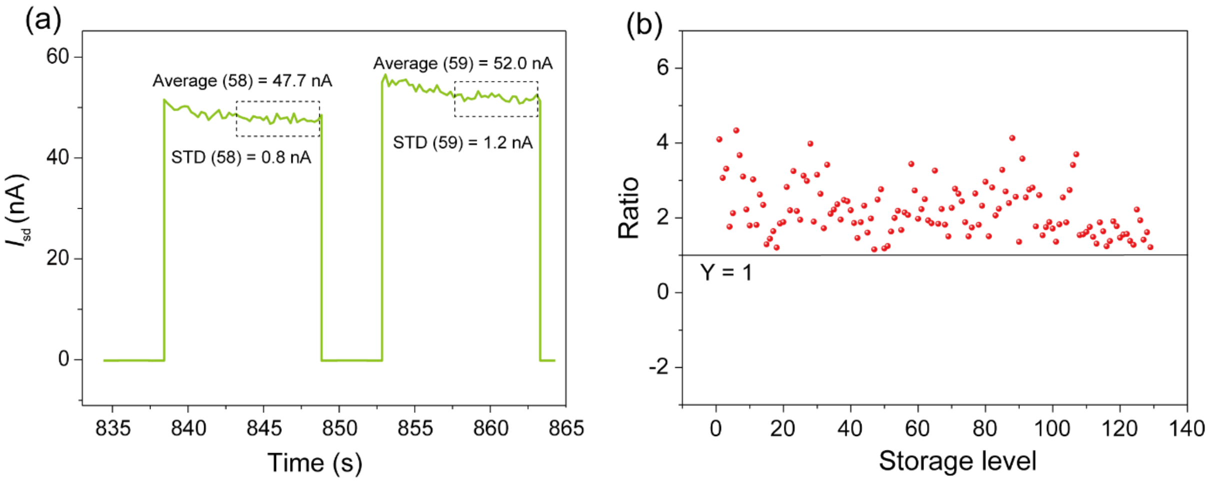

Supplementary Figure 7 | Noise calculations for reliable storage states. (a) Storage currents and the STDs of states 58 and 59. **(b)** Gap to STD ratio for all the storage states.

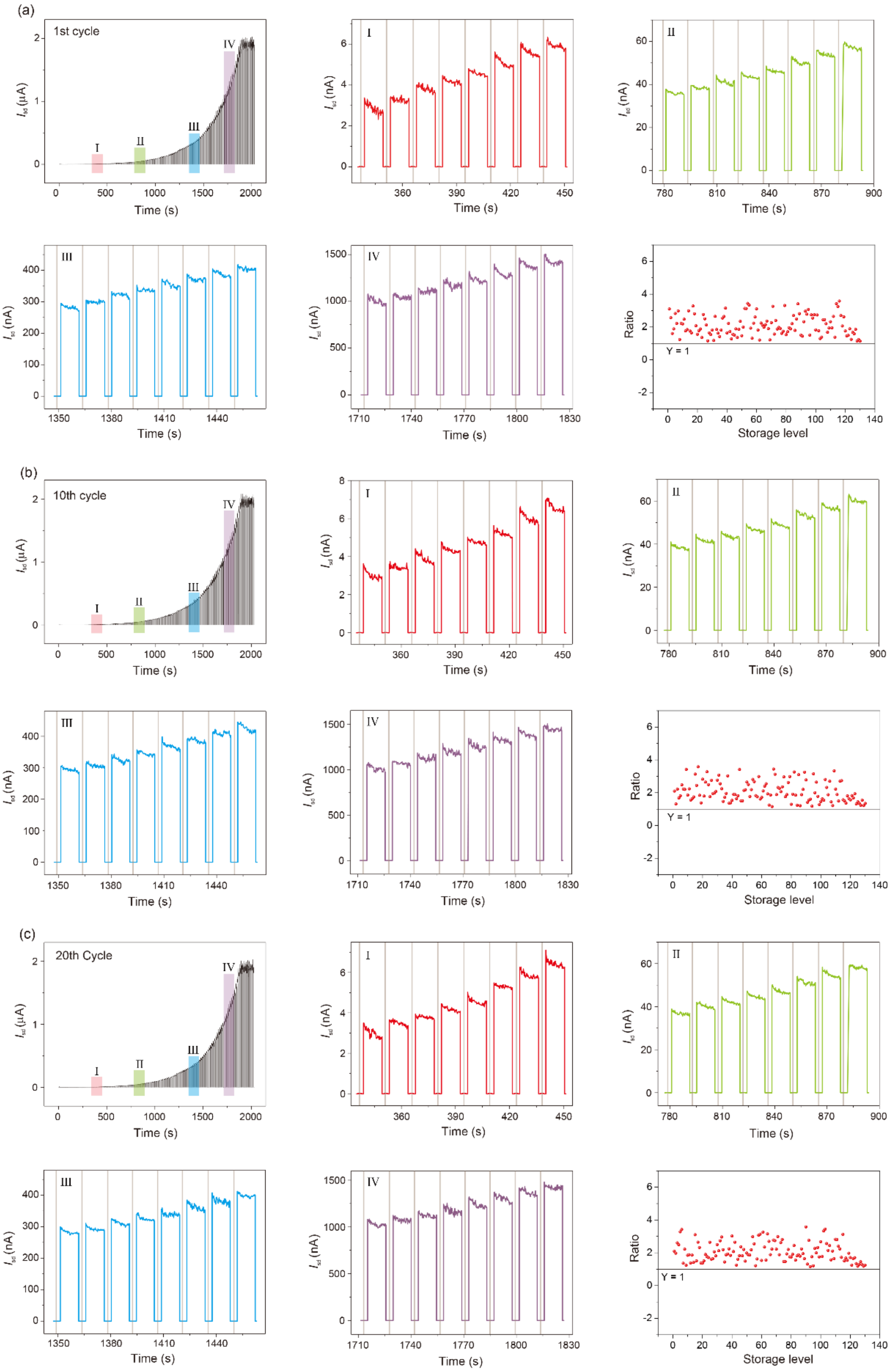

Supplementary Figure 8 | Repeatability of the memory. Dynamic behavior and the gap to STD sum ratios of the memory in the 1st **(a)**, 10th **(b)**, and 20th **(c)** cycle.

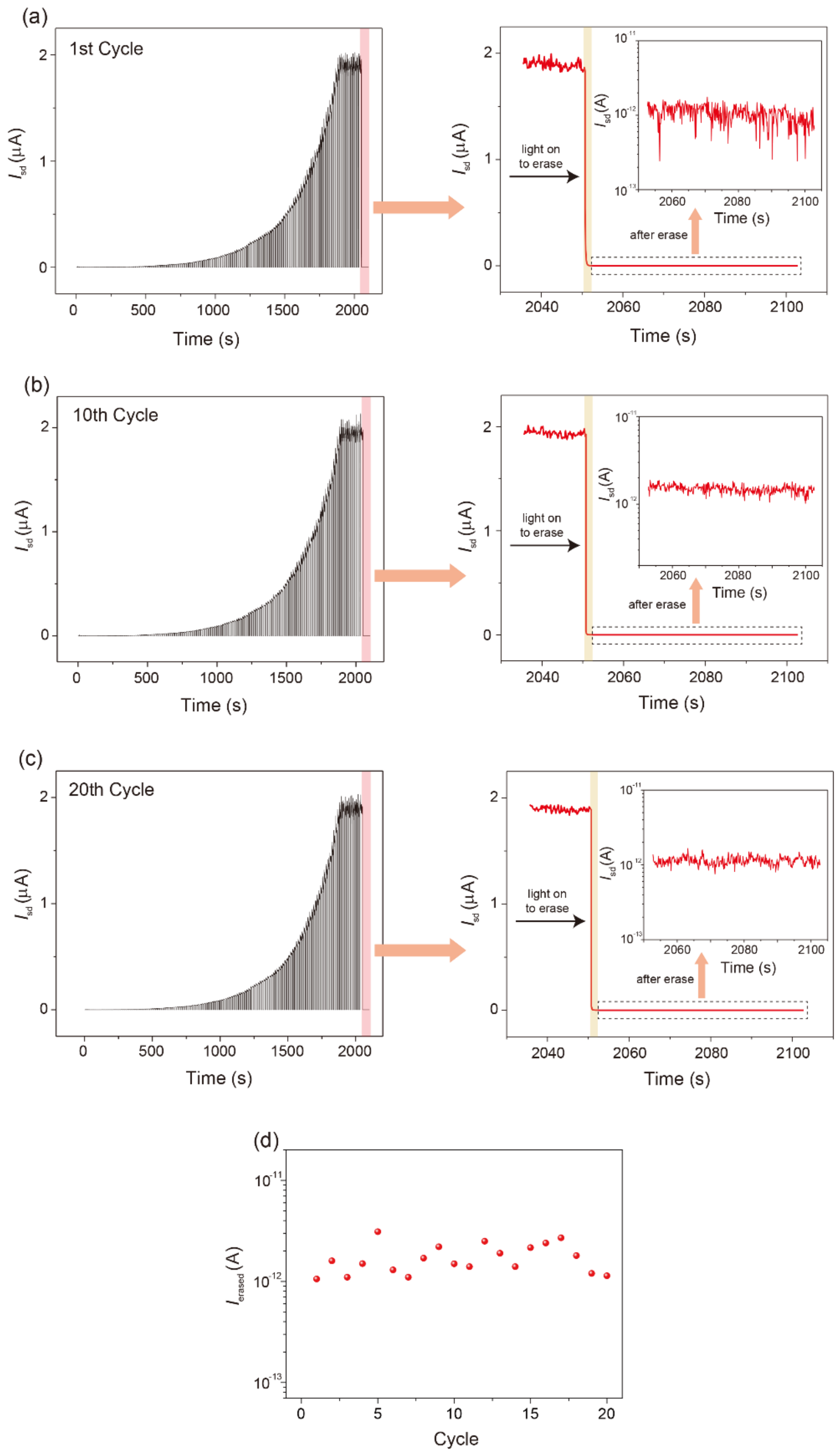

Supplementary Figure 9 | Erased currents of the memory. Erased current for the 1st **(a)**, 10th **(b)**, and 20th **(c)**. **(d)** Summary of the erased currents for the 20 independent cycles.

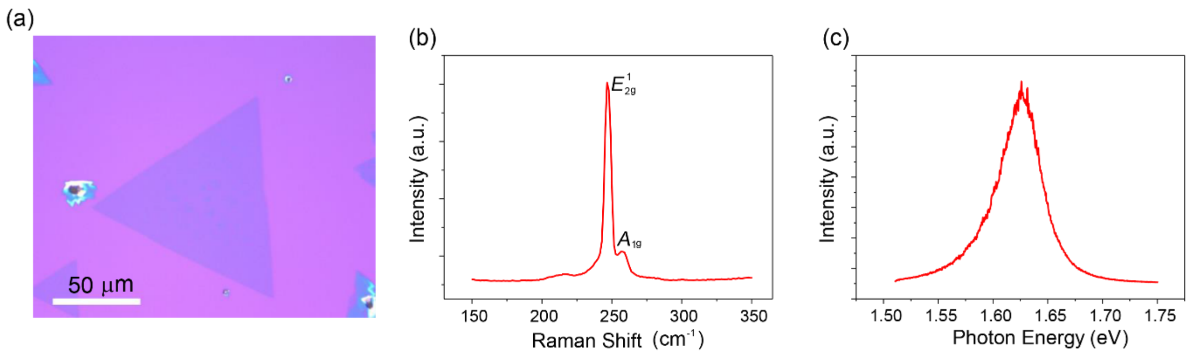

Supplementary Figure 10 | Raman and PL characterizations of CVD grown large area WSe_2. Optical image **(a)**, Raman **(b)**, and PL **(c)** of the CVD WSe_2. Supplementary Fig.10a shows the optical image of CVD grown WSe_2 in triangle shape with scale around 100 μm. Both the Raman and PL spectra confirm the monolayer characteristic of the CVD WSe_2.

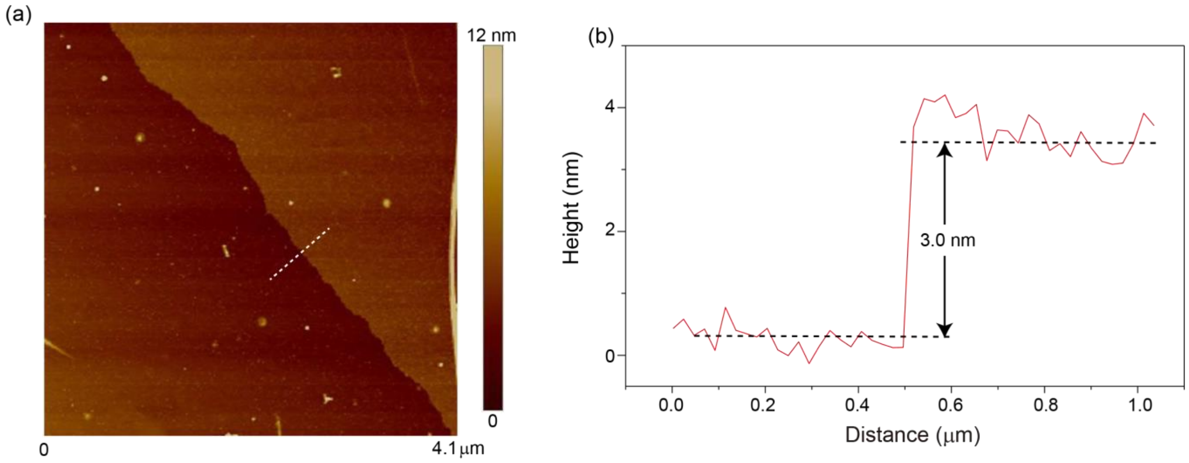

Supplementary Figure 11 | Thickness variation of the BN substrate in WSe_2/BN pixel matrix. AFM image **(a)** and its corresponding line profile **(b)** of the BN substrate at the interface of the bright and dark regions in WSe_2/BN pixel matrix SEM image. The bright and dark zones are clearly observed in the false-colored SEM image of the fabricated WSe_2/BN pixel matrix, which is due to the thickness variation (approximating 3 nm) of the BN substrate.

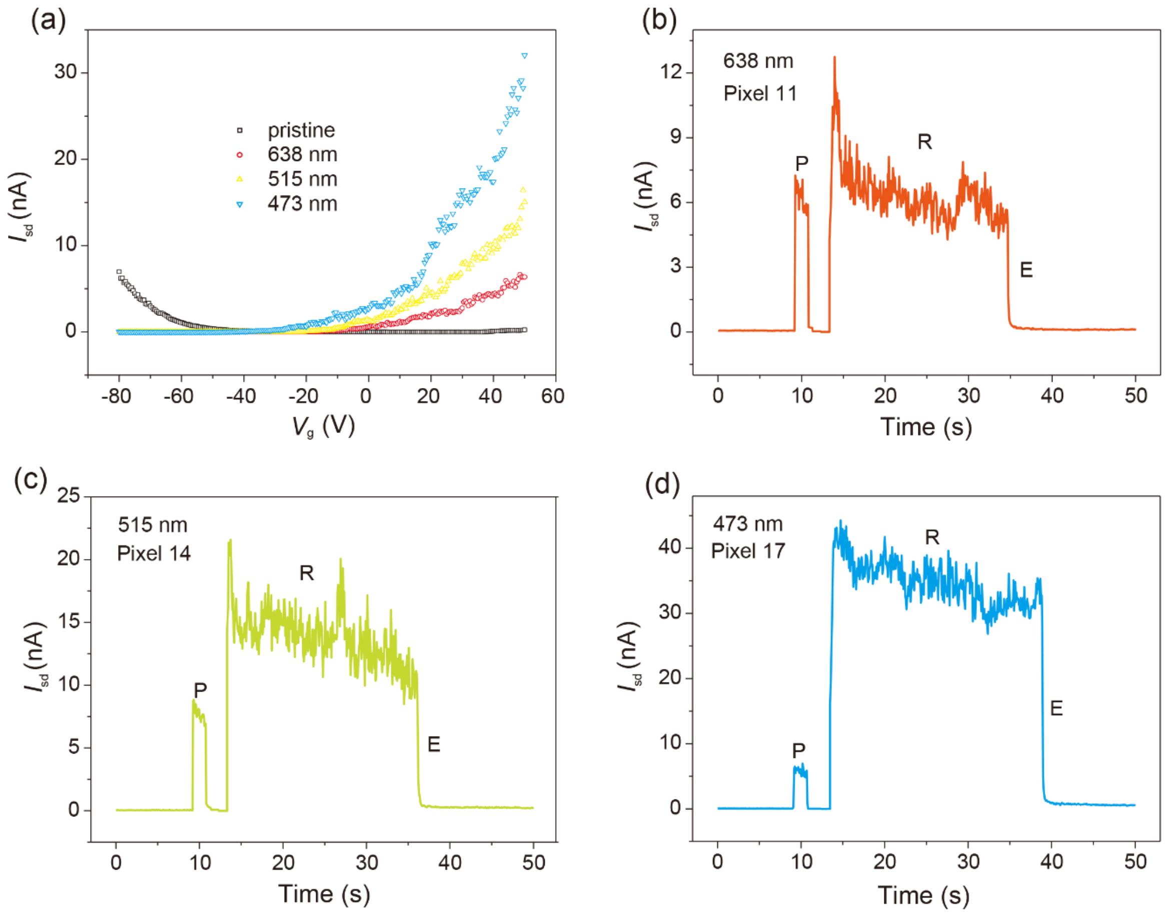

Supplementary Figure 12 | Storage states of the selected pixels under different wavelength lights. Transfer characteristics **(a)** and the corresponding dynamic behaviors of the three selected pixels (11, 14, and 17) under red 638 nm **(b)**, green 515 nm **(c)**, and blue 473 nm **(d)** lights respectively. P, R, and E refer to program, readout, and erase respectively. As shown in Supplementary Fig.12a, the on current at the electron regime gradually rises with reducing light wavelength, which is consistent with the dynamic behavior in Supplementary Fig.12b-12d. The storage currents for the selected pixels are around 5.2 nA, 12.8 nA, and 31.5 nA under the wavelengths of 638 nm, 515 nm, and 473 nm respectively.

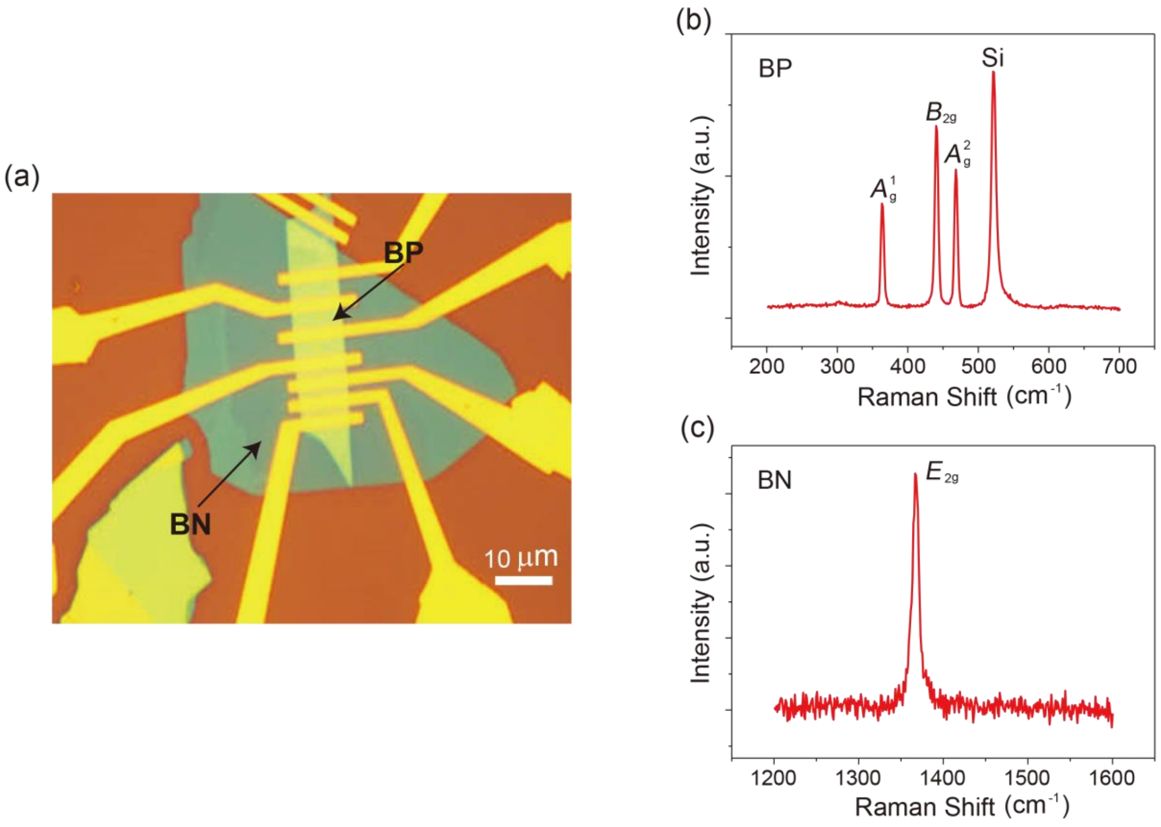

Supplementary Figure 13 | Raman spectra of BP and BN crystals. (a) Optical image of the BP/BN heterostructure. Raman spectra of the exfoliated BP **(b)** and BN **(c)** flakes. The first order Raman spectrum of exfoliated few-layer BP exhibits the characteristic peaks nearly located at 363 cm^{-1}, 440 cm^{-1}, and 468 cm^{-1}, corresponding to the three different vibration modes A_g^1, B_{2g}, and A_g^2 in BP crystal lattice, respectively[2]. The position of the characteristic peak for BN flake is located at 1367 cm^{-1}, almost the same as the position of the BN flake measured in WSe_2/BN structure above.

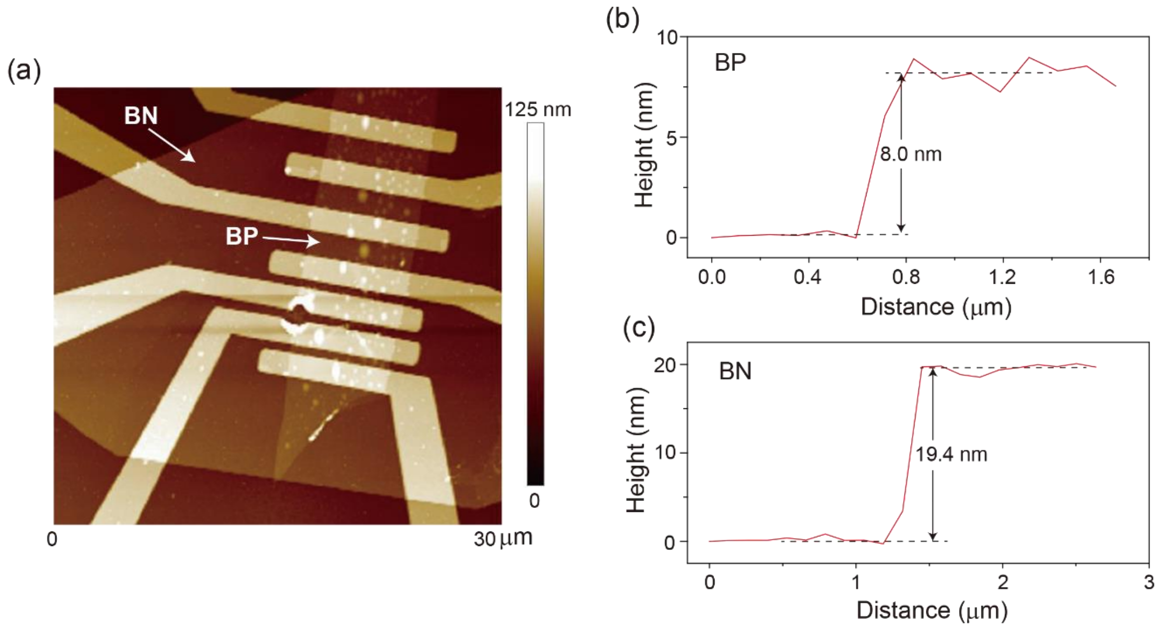

Supplementary Figure 14 | AFM images of BP and BN flakes. (a) AFM image of the as-fabricated BP/BN memory device. The height profiles of BP **(b)** and BN **(c)** flakes. The thickness of BP and BN flakes are 8.0 nm and 19.4 nm, corresponding to the layer numbers of 13 and 39, respectively.

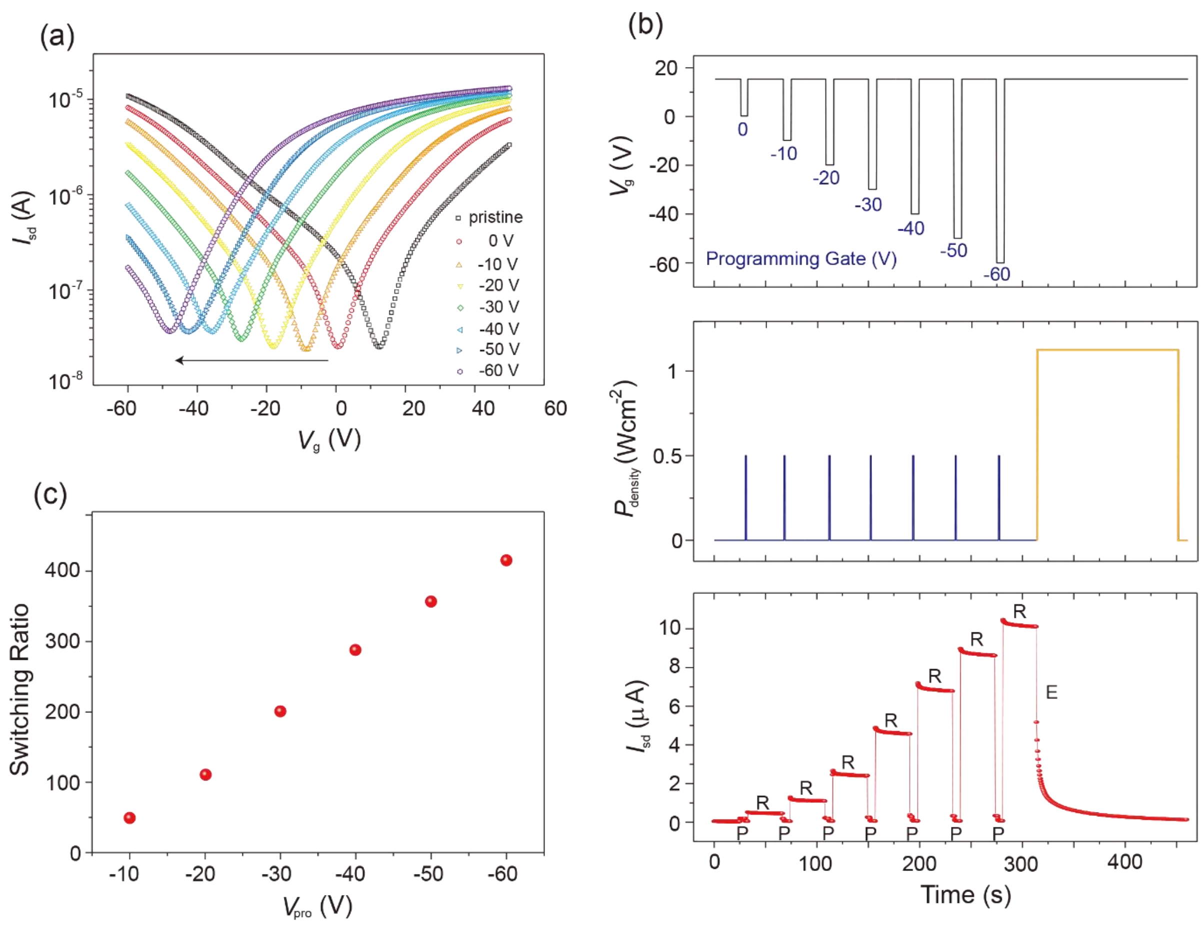

Supplementary Figure 15 | Gate controlled BP/BN memory. (a) Transfer characteristics evolution of the BP/BN memory with respect to V_{pro} at V_{sd} = 0.1 V. The light pulse is the same for each programming process with intensity 10 mW cm^{-2} and dwell time 200 s. **(b)** Gate dependent dynamic behavior of the BP/BN memory. The storage current is readout and erased at V_g = 15 V since the current minimum of the pristine BP/BN FET is around 15 V. **(c)** Plot of switching ratio as a function of programming gate.

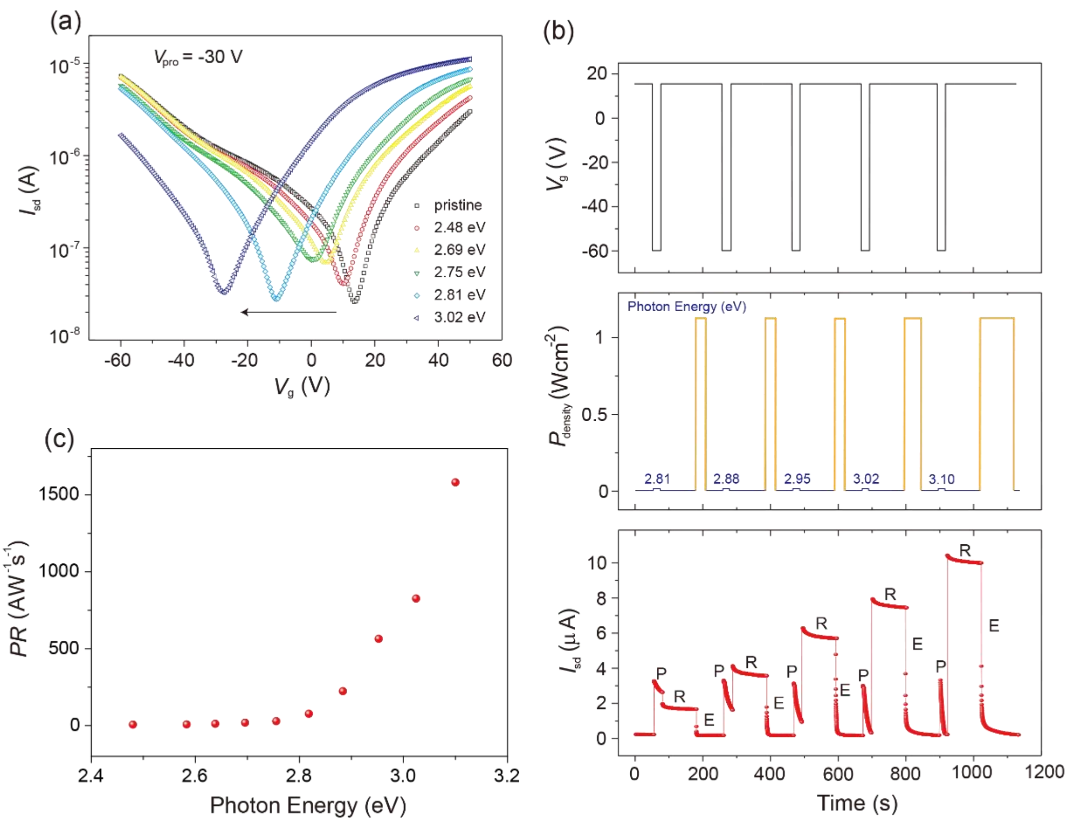

Supplementary Figure 16 | Wavelength discrimination of BP/BN memory. (a) Transfer characteristics evolution of the BP/BN memory with respect to photon energy from 2.48 eV to 3.02 eV at V_{sd} = 0.1 V, V_{pro} = -30 V. The programming time is the same for each process. **(b)** Wavelength controlled dynamic behavior of the BP/BN memory. **(c)** Programming rate versus photon energy. The transfer curve progressively moves towards negative gate voltage with increasing the photon energy, suggesting the greater number of positive charges generated in BN, same as the effect observed in WSe_2/BN memory. This result is consistent with the dynamic behavior shown in Supplementary Fig.16b. When increasing the photon energy from 2.81 eV to 3.10 eV, 5 distinct storage states are clearly identified. The *PR* increases slowly at the photon energy smaller than 2.8 eV, before a sharp rise with the continual increase of the photon energy. This photon energy dependent property is similar to that of the WSe_2/BN memory, which is due to the distribution of donor-like states in BN material.

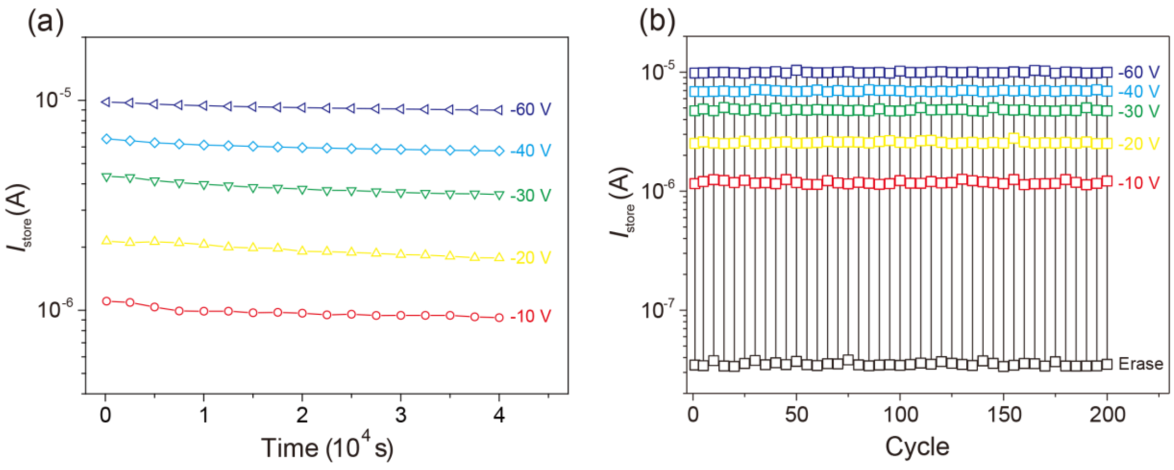

Supplementary Figure 17 | Data reliability tests for BP/BN memory. Data retention **(a)** and cyclic endurance **(b)** of the BP/BN memory. The tests were taken at 5 different V_{pro} from -10 V to -60 V with -10 V step. The black squares represent the device in the erased state, while the symbols in red, yellow, green, blue, and purple represent the programming gate -10V, -20V, -30V, -40V, and -60V respectively. The storage currents after each programming nearly remain in the time range of 4×10^4 s, which indicates the excellent data retention property of the BP/BN memory. Moreover, the fluctuations in the cyclic tests are quite small, suggesting that the programmed data in the BP/BN memory is highly reproducible.

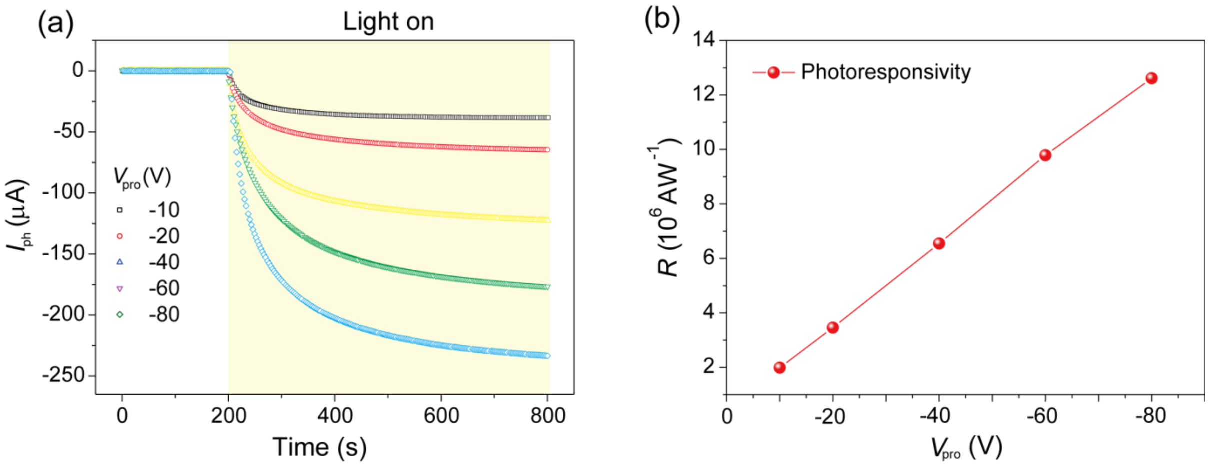

Supplementary Figure 18 | Photoresponse of BP/BN photodetector. (a) Time dependence of photocurrent (I_{ph}) measured at different programming gates (V_{pro} = -10 V, -20 V, -40 V, -60 V, -80 V). V_{sd} = 1 V for all the photoresponse measurements. **(b)** Calculated photo responsivity as a function of the V_{pro}.

Supplementary Note 1 | Raman and PL spectra of the exfoliated WSe_2 and BN Samples

Supplementary Fig. 1a shows the Raman spectrum of the exfoliated WSe_2, with two characteristic peaks located at 250 cm^{-1} and 261 cm^{-1} respectively. There is no distinct peak at 316 cm^{-1}, indicating the monolayer nature of the WSe_2 flake[3]. This result is further confirmed by the PL spectrum (Supplementary Fig. 1b) which exhibits a strong PL peak at 1.65 eV, belonging to single-layer WSe_2[4]. The Raman mapping of E^1_{2g} mode indicates the high uniformity of the monolayer WSe_2 flake (Supplementary Fig.1c). Supplementary Fig.1d illustrates the Raman spectrum of the BN flake, with one characteristic peak at 1365 cm^{-1}.

Supplementary Note 2 | Saturation of the storage current

In our experiment, we observed that the storage current gradually saturates with increasing the pulse number, which is shown in Supplementary Fig.6a. The storage current almost maintains when the storage level is more than 130 (Supplementary Fig.6b). In our WSe_2/BN memory, the photon-excited electrons in BN conduction band can transfer into WSe_2 driven by the electric field, leaving the positive charges localized in middle of the BN bandgap. It is worth noting that these localized positive charges in BN can effectively screen the negative gate and hence weaken the electric field exerting on WSe_2 during the programming process. The elimination of the effective electric field in BN symbolizes the termination of the programming process and results in the saturation of the storage current.

Supplementary Note 3 | Noise calculations for reliable storage states

Supplementary Fig.7a shows an example about the calculation of the storage currents and standard deviations (STDs) for states 58 and 59. When the gate voltage is switched to 50 V after programming, the electron-domination current increases sharply followed by stabilization. Both the average storage currents and their STDs are calculated in the area where the current becomes stable (encircled by a rectangle in Supplementary Fig.7a). The storage currents of states 58 and 59 are 47.7 nA and 52.0 nA, while their STDs are 0.8 nA and 1.2 nA respectively. The gap between states 58 and 59 should be larger than their STD sums, so that these two states are distinguishable. Therefore, we define the gap to STD sum ratio below:

$$Ratio = \frac{I_{\text{store}}(n+1) - I_{\text{store}}(n)}{STD(n+1) + STD(n)} \quad (1)$$

Where n = 1, 2, 3…., 129. Through substituting the storage currents and STDs of states 58 and 59 in the formula, we derive the ratio of 2.15, larger than 1, indicating that these two states are distinguished from each other. The reliability of the other states is evaluated by the same method, where the result is plotted in Supplementary Fig.7b. All the ratios are beyond the critical line Y = 1, which suggests the validity of all the storage states.

Supplementary Note 4 | Gate controlled BP/BN memory

The initial transfer curve presents a current minimum of 15 V. When increasing the V_{pro} to -60 V, the current minimum progressively moves to -48 V. This suggests a significant electron-doping effect in BP modulated by backgate, a characteristic similar to that of the WSe_2/BN memory. The dynamic behavior shows that the storage current increases stepwise with the increase of V_{pro}, consistent with the transfer characteristics evolution. 7 distinguishable storage states are observed in Supplementary Fig.15b through applying 7 different V_{pro}. The switching ratio rises in a nearly linear trend with respect to V_{pro}, from 49 to 415 (Supplementary Fig.15c).

It should be noted that the erasing time of the BP/BN memory (150 s) is longer comparing to that of the WSe_2/BN memory. We propose that the long erasing time in BP/BN memory is mainly due to the electron trapping states in BP which was studied before[5,6]. During programming, the photogenerated electrons in BN can transfer and store in BP driven by the external electric field. Some of these transferred electrons could be trapped by the trapping sites inside BP. In the erasing process, the ionized positive defects are filled by photon-excited electrons from BN valence band, generating large quantity of holes. Attributing to the external electric field induced by the positive gate, the generated holes in BN and the stored electrons in BP accumulate at the BP/BN interface. Consequently, the electron-hole pairs intensely recombine, which leads to the erase of stored electrons in BP. However, those trapped electrons in BP are quite localized, which makes them difficult to be recombined, therefore, resulting in the long erasing time. In the case of WSe_2, the shorter erasing time is expected due to the absence of locally trapped electrons.

Supplementary Note 5 | Photoresponse of BP/BN photodetector

I_{ph} is defined as the current difference of the BP/BN device under dark and light illumination conditions: $I_{ph} = I_{light} - I_{dark}$. The photocurrent is strongly dependent on V_{pro}, where the I_{ph} gradually increases with the increase of gate voltage. The photo responsivity (R) as a critical parameter of photodetector performance, is calculated and plotted as a function of V_{pro} as shown in Supplementary Fig.18b. R is defined as the photocurrent generated by per unit power of incident light on the effective area of a photodetector[5]:

$$R = I_{ph} / (P_{density} \cdot S) \quad (2)$$

The photo responsivity displays nearly linear dependence on the programming gate, progressively increasing from 2×10^{6} AW^{-1} (V_{pro} = -10 V) to 1.2×10^{7} AW^{-1} (V_{pro} = -80 V). This extraordinary photo responsivity is comparable to or even larger than the results of recently reported ultrasensitive BP photodetectors[7,8], indicating BP/BN heterostructure as a promising candidate for photodetector application.

Supplementary References

1 Ju, L., Jr, J. V., Huang, E., Kahn, S., Nosiglia, C., Tsai, H., Yang, W., Taniguchi, T., Watanabe, K., Zhang, Y., Zhang, G., Crommie, M., Zettl, A., Wang, F. Photoinduced doping in heterostructures of graphene and boron nitride. *Nat. Nano.*, **9**, 348-352 (2014).

2 Sugai, S. & Shirotani, I. Raman and infrared reflection spectroscopy in black phosphorus. *Solid State Commun.*, **53**, 753-755 (1985).

3 Zhao, W., Ghorannevis, Z., Amara, K. K., Pang, J. R., Toh, M., Zhang, X., Kloc, C., Tang, P. H., Eda, G., Lattice dynamics in mono-and few-layer sheets of WS_2 and WSe_2. *Nanoscale,* **5**, 9677-9683 (2013).

4 Zeng, H., Liu, G., Dai, J., Yan, Y., Zhu, B., He, R., Xie, L., Xu, S., Chen, X., Yao, W., Cui, X., Optical signature of symmetry variations and spin-valley coupling in atomically thin tungsten dichalcogenides. *Sci. Rep.*, **3**, 1608-1613 (2013).

5 Xiang, D., Han, C., Wu., J., Zhong, S., Liu, Y., Zhang, X., Hu, W., Ozyilmaz, B., Neto, A. H. C., Wee, A. T. S., Chen, W. Surface transfer doping induced effective modulation on ambipolar characteristics of few-layer black phosphorus. *Nat. Commun.*, **6**, 6485-6493 (2015).

6 Doganov, R. A., O'Farrell, E. C. T., Koenig, S. P., Yeo, Y., Ziletti, A., Carvalho, A., Campbell, D. K., Watanabe, K., Taniguchi, T., Neto, A. H. C., Ozyilmaz, B., Transport properties of pristine few-layer black phosphorus by van der Waals passivation in an inert atmosphere. *Nat. Commun.*, **6**, 6647-6654 (2015).
7 Huang, M., Wang, M., Chen, C., Ma, Z., Li, X., Han, J., Wu, Y., Broadband Black-Phosphorus Photodetectors with High Responsivity. *Adv. Mater.*, **28**, 3481-3485 (2016).
8 Liu, F., Zhu, C., You, L., Liang, S., Zheng, S., Zhou, J., Fu, Q., He, Y., Zeng, Q., Fan, H., Ang, L. K., Wang, J., Liu, Z., 2D Black Phosphorus/$SrTiO_3$-Based Programmable Photoconductive Switch. *Adv. Mater.*, **28**, 7768-7773 (2016).
